# Influence of diet and physical exercise on endometriosis: a systematic review

**DOI:** 10.15649/cuidarte.5426

**Published:** 2026-04-30

**Authors:** Alba Fuentes Rodríguez, Óscar Grela Beres

**Affiliations:** 1 Clinical nurse, Galician Health Service, Santiago de Compostela, Spain. E-mail: alba.fuentes.rodriguez@sergas.es Galician Health Service Santiago de Compostela Spain alba.fuentes.rodriguez@sergas.es; 2 Family and community medicine physician, Galician Health Service, Santiago de Compostela, Spain. E-mail: oscar.grela.beres@sergas.es Galician Health Service Santiago de Compostela Spain oscar.grela.beres@sergas.es

**Keywords:** Endometriosis, Exercise, Diet, Quality of Life, Systematic Review, Endometriosis, Ejercicio Físico, Dieta, Calidad de Vida, Revisión Sistemática, Endometriose, Exercício Físico, Dieta, Qualidade de Vida, Revisão Sistemática

## Abstract

**Introduction:**

Endometriosis is a chronic inflammatory disease that affects 10% of women, causing pelvic pain, dysmenorrhea, and infertility, with a strong impact on quality of life.

**Objective:**

To analyze the current scientific evidence on the influence of diet and physical exercise on symptoms and quality of life in women of reproductive age with endometriosis.

**Materials and Methods:**

A systematic review of studies published between 2014 and 2024 was conducted using the descriptors endometriosis, exercise, diet, and quality of life in the following databases: MEDLINE, Web of Science, Scopus, SciELO, Virtual Health Library, Dialnet, and Cochrane Library. Systematic reviews, meta-analyses, case reports, letters, and articles without full-text access were excluded. Two independent reviewers performed study selection and data extraction, and methodological quality was assessed according to study design.

**Results:**

Of 523 initial records, 13 articles were included (7 on diet and 6 on exercise). Anti-inflammatory dietary interventions and regular physical exercise were associated with reduced symptoms and improved quality of life.

**Discussion:**

Diet and exercise may influence the symptoms and well-being of these patients, although the evidence is limited and inconsistent. The included studies show methodological heterogeneity, small sample sizes, and variability in interventions and outcomes, limiting internal validity and the generalizability of the findings.

**Conclusion:**

Diet and exercise constitute complementary strategies to pharmacological and/or surgical treatment within a comprehensive approach to endometriosis; however, more rigorous research is needed to support their long-term benefits.

## Resumen

**Introducción:**

La endometriosis es una enfermedad inflamatoria crónica que afecta al 10% de las mujeres, provocando dolor pélvico, dismenorrea e infertilidad, con un impacto significativo en la calidad de vida.

**Objetivo:**

Analizar la evidencia científica actual sobre la influencia de la dieta y ejercicio físico, en los síntomas y la calidad de vida de mujeres en edad fértil con endometriosis.

**Materiales y métodos:**

Se realizó una revisión sistemática de estudios publicados entre 2014 y 2024, usando los descriptores endometriosis, exercise, diet y quality of life, en las siguientes bases de datos: Medline, Web of Science, Scopus, Scielo, Biblioteca Virtual de Salud, Dialnet y Cochrane. Se excluyeron revisiones sistemáticas, metaanálisis, casos clínicos, cartas y artículos sin acceso completo. Dos revisores independientes realizaron selección y extracción de datos, evaluando la calidad metodológica según el tipo de estudio.

**Resultados:**

De 523 registros iniciales, se seleccionaron 13 artículos (7 sobre dieta y 6 sobre ejercicio). Intervenciones dietéticas antiinflamatorias y ejercicio físico regular se asociaron con reducción de síntomas y mejoría en la calidad de vida.

**Discusión:**

Dieta y ejercicio pueden influir en los síntomas y bienestar de estas pacientes, aunque la evidencia es limitada e inconsistente. Los estudios presentan heterogeneidad metodológica, muestras pequeñas y variabilidad en las intervenciones y resultados, limitando la validez interna y la generalización de los hallazgos.

**Conclusiones:**

Dieta y ejercicio constituyen estrategias complementarias al tratamiento farmacológico y/o quirúrgico dentro de un abordaje integral de la endometriosis, si bien se precisa investigación más rigurosa que respalde sus beneficios a largo plazo.

## Resumo

**Introdução:**

A endometriose é uma doença inflamatória crónica que afeta 10% das mulheres, provocando dor pélvica, dismenorreia e infertilidade, com forte impacto na qualidade de vida.

**Objetivo:**

Avaliar as evidências científicas atuais sobre a influência da dieta e do exercício físico nos sintomas e na qualidade de vida de mulheres em idade fértil com endometriose.

**Materiais e métodos:**

Foi realizada uma revisão sistemática de estudos publicados entre 2014 e 2024, utilizando os descritores endometriose, exercício físico, dieta e qualidade de vida, nas seguintes bases de dados: Medline, Web of Science, Scopus, SciELO, Biblioteca Virtual em Saúde, Dialnet e Cochrane. Revisões sistemáticas, meta-análises, relatos de caso, cartas e artigos sem acesso completo foram excluídos. Dois revisores independentes realizaram a seleção e extração de dados, avaliando a qualidade metodológica de acordo com o tipo de estudo.

**Resultados:**

De 523 registros iniciais, 13 artigos foram selecionados (7 sobre dieta e 6 sobre exercício físico). Intervenções dietéticas anti-inflamatórias e exercícios físicos regulares foram associados à redução dos sintomas e à melhora da qualidade de vida.

**Discussão:**

Dieta e exercícios podem influenciar os sintomas e o bem-estar dessas pacientes, embora as evidências sejam limitadas e inconsistentes. Os estudos apresentam heterogeneidade metodológica, amostras pequenas e variabilidade nas intervenções e nos desfechos, o que limita a validade interna e a generalização dos resultados.

**Conclusões:**

Dieta e exercícios são estratégias complementares ao tratamento farmacológico e/ou cirúrgico dentro de uma abordagem abrangente para a endometriose, embora sejam necessárias pesquisas mais rigorosas para comprovar seus benefícios a longo prazo.

## Introduction

Endometriosis is a chronic, estrogen-dependent inflammatory disease characterized by the presence of endometrial-like tissue outside the uterine cavity. Its etiology is multifactorial, involving genetic, immunological, and environmental factors^1^-^3^. It affects approximately 10% of women of reproductive age worldwide^4^,^5^, with a significant impact on physical and emotional health and quality of life^4^. Its prevalence varies across regions due to differences in health systems, cultural factors, and socioeconomic conditions. In Colombia, population-based studies are lacking; however, patient associations report that approximately 3.5 million women are affected^6^.

Clinically, chronic pelvic pain, severe dysmenorrhea, and dyspareunia predominate, occurring in up to 75% of cases. Menstrual disorders and infertility are also common, with infertility affecting up to 40% of diagnosed women^1^,^2^,^4^,^7^. On physical examination, findings may include a retroverted (tilted) uterus, pelvic tenderness, and enlarged ovaries^6^. Pain and infertility are key symptoms guiding the diagnostic assessment^2^,^7^.

Endometriosis is classified according to lesion location and depth of infiltration: superficial (type I), characterized by small lesions; ovarian (type II), characterized by endometriomas; and deep infiltrating endometriosis (type III), characterized by nodules >5 mm^5^,^7^. Diagnosis is based on medical history, physical examination, and imaging modalities (such as ultrasound or magnetic resonance imaging), although definitive diagnosis remains laparoscopic7. Factors such as family history, early menarche, and short menstrual cycles are associated with increased risk^7^.

Endometriosis management requires a multidisciplinary approach aimed at relieving pain, preserving fertility, and improving quality of life. There is no curative treatment; management focuses on slowing disease progression and controlling symptoms through pharmacological, surgical, or combined approaches^4^,^7^,^8^. Hormonal therapies (combined oral contraceptives, progestins, and gonadotropin- releasing hormone [GnRH] analogs) temporarily reduce estrogenic stimulation of ectopic tissue, and surgery is indicated only in refractory cases or when malignancy is suspected^7^.

Proinflammatory diets, physical inactivity, and low antioxidant intake have been associated with more severe disease^2^,^8^-^10^. Diet may modulate estrogen levels, promote a healthy gut microbiota, and reduce proinflammatory prostaglandins and cytokines^11^-^13^. Likewise, antioxidant supplementation may reduce symptoms^10^.

Physical exercise is defined as planned and repetitive bodily activity performed to improve physical fitness^14^. In animal models, reductions in proinflammatory myokines have been observed following training^15^. Regular exercise may decrease cytokines such as TNF-α and IL-6, as well as circulating estrogen levels, thereby reducing stimulation of ectopic tissue and attenuating inflammation^16^,^17^. Pelvic floor muscle training has traditionally been recommended for women with chronic pelvic pain^5^, and recent studies suggest modulatory effects on pain and inflammation^14^,^15^.

Dietary habits and physical activity may have a positive effect on symptom control and quality of life in women with endometriosis by modulating inflammatory, hormonal, and immunological processes associated with the disease. This review aims to analyze how lifestyle modifications, particularly in diet and physical exercise, may contribute to symptom control and improved quality of life in women with endometriosis.

## Materials and Methods

A systematic review was conducted in accordance with the PRISMA 2020 statement (Preferred Reporting Items for Systematic Reviews and Meta-Analyses). This review was not registered. As this study is a systematic review of previously published research, ethics committee approval was not required. No individual patient data or confidential information was used.

The research question was formulated according to the PICO framework Table 1 and aims to assess whether, among women of reproductive age diagnosed with endometriosis, dietary patterns and physical exercise are associated with differences in symptoms and quality of life, beyond standard symptomatic treatment.

Original studies published in English, Spanish, French and Portuguese between 2014 and 2024 that addressed diet or physical exercise in patients with a diagnosis of endometriosis were included. Reviews, case reports, or case series, letters to the editor, conference abstracts, and articles without full-text access were excluded.

**Table 1:** PICO Framework

P (population)	Women of reproductive age with a confirmed diagnosis of endometriosis
I (intervention)	Patients’ dietary habits and food patterns Patients’ physical activity
C (comparison)	Conventional pharmacological and/or surgical treatment of the disease
O (Outcomes)	Patients’ symptoms Women’s quality of life

The literature search was conducted in MEDLINE, Web of Science (WOS), Scopus, SciELO, Virtual Health Library, Dialnet, and the Cochrane Library. The search strategy included MeSH terms (“endometriosis,” “exercise,” “diet,” and “quality of life”) and DeCS descriptors (“endometriosis,” “diet,” “physical activity,” and “quality of life”), combined using Boolean operators (AND and OR) Table 2. The last search was performed on December 18, 2024.

**Table 2:** Search Strategy Across Databases

MEDLINE	(("Endometriosis"[Mesh]) AND ("Exercise"[Mesh] OR "Diet"[Mesh])) (("Endometriosis"[Mesh]) AND ("Exercise"[Mesh] OR "Diet"[Mesh]) AND ("Quality of Life"[Mesh])) Filters: Clinical Study, Clinical Trial, Multicenter Study, Observational Study; Publication dates: 2014/01/01–2024/12/31
Web of Science	TS=("Endometriosis") AND TS=("Exercise" OR "Diet") AND TS=("Quality of Life") Refined by: Document Types = (ARTICLE OR CLINICAL TRIAL) Timespan: 2014–2024
SciELO	(“Endometriosis”) AND (“dieta” OR “ejercicio físico” OR “deporte”) (“Endometriosis”) AND (“dieta” OR “ejercicio físico” OR “deporte”) AND (“calidad de vida”) Filtered by: Articles; Publication years:2014–2024
Scopus	(TITLE-ABS-KEY("Endometriosis") AND TITLE-ABS-KEY("Exercise" OR "Diet") (TITLE-ABS-KEY("Endometriosis") AND TITLE-ABS-KEY("Exercise" OR "Diet") siAND TITLE-ABS-KEY("Quality of Life")) Filtered by: Article; Publication years 2014–2024
Cochrane Library	("Endometriosis" in Title Abstract Keyword) AND ("Exercise" OR "Diet" in Title Abstract Keyword) Filtered by: Trials; Publication years 2014–2024
Virtual Health Library and Dialnet	(Endometriosis) AND (“Ejercicio físico” OR “Dieta” OR "Calidad de vida") Filters: Publication years: 2014–2024

Two independent reviewers selected the studies in two stages: title and abstract screening, followed by full-text review of potentially eligible articles. Discrepancies were resolved by consensus. Zotero was used for reference management and duplicate removal, and the Rayyan platform facilitated study selection between reviewers. A standardized data extraction form was subsequently developed to collect relevant information from each study, including authors, year of publication, study design, sample characteristics, objectives, intervention and comparison group, and main outcomes.

Methodological quality and risk of bias were assessed using the Effective Public Health Practice Project Quality Assessment Tool (EPHPP) for quantitative studies and the Consolidated Criteria for Reporting Qualitative Research (COREQ) for qualitative research. According to the EPHPP, studies were classified as having strong, moderate, or weak methodological quality, while for the COREQ checklist, studies with <50% item adherence were considered low quality and those with ≥75% adherence were considered high quality^18^,^19^. Discrepancies were resolved by consensus.

A descriptive analysis of the extracted data was conducted. The results are presented narratively and in tables, including relevant information from each study. All collected data are publicly available in Mendeley Data^20^.

## Results

**Figure 1:**
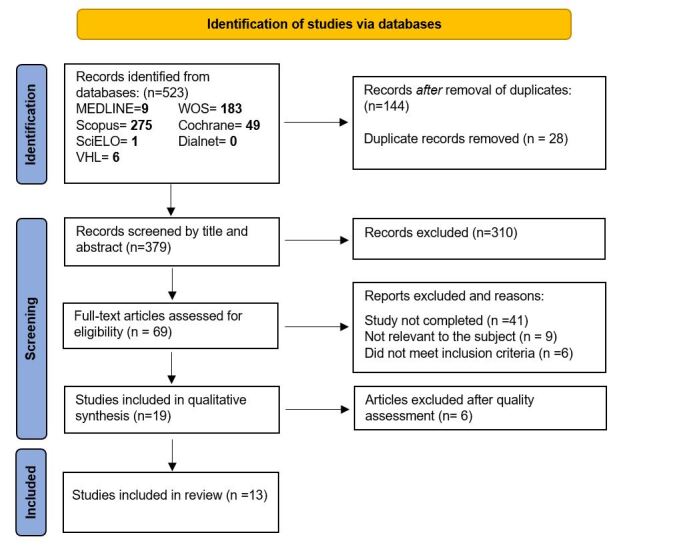
Flow diagram of study selection

The initial search yielded a total of 523 records from databases including MEDLINE, Web of Science, Scopus, SciELO, the Virtual Health Library, and the Cochrane Library. After removal of duplicates (n = 144), an additional 310 records were excluded following title and abstract screening. A total of 69 articles were selected for full-text assessment Figure 1. This systematic review ultimately included 13 studies published between 2021 and 2024, encompassing various study designs: randomized controlled trials (n = 3), one quasi-experimental study (n = 1), observational studies (n = 8), and one qualitative study. Six studies focused on physical exercise-related outcomes, while seven addressed dietary habits or interventions. The main characteristics of these studies are presented in Table 3.

Several studies reported a positive association between dietary modification and improvement in symptom control or quality of life in women with endometriosis. Van Haaps et al.^21^ demonstrated, in a nonrandomized experimental study, that adherence to an endometriosis-specific diet or a low- FODMAP diet (low in fermentable oligosaccharides, disaccharides, monosaccharides, and polyols) over six months significantly reduced symptoms such as deep dyspareunia, dysuria, bloating, and fatigue. Improvements were also observed across multiple quality-of-life domains, including pain, functional limitation, emotional well-being, self-image, work life, and sexual relationships. Additionally, significant improvement in gastrointestinal health was observed, as assessed using the Gastrointestinal Quality of Life Index (GIQLI)^22^, whereas the control group showed no relevant changes.

Similarly, Ghasemisedaghat et al.^2^, in a case-control study, reported that higher adherence to the so-called “fertility diet,” characterized by high intake of plant protein, monounsaturated fats, and multivitamins, and low intake of animal protein, heme iron, and glycemic load, was associated with a significantly lower likelihood of endometriosis, suggesting a protective effect of certain dietary patterns. The likelihood of endometriosis was 82% and 69% lower in adjusted models with higher intake of plant protein and multivitamins, respectively. In contrast, high intake of animal protein, heme iron, and higher glycemic load were positively associated with an increased likelihood of endometriosis.

Other observational studies, such as van Haaps et al.^4^, showed that women who adhered to an “endometriosis diet,” characterized by the elimination of red meat, gluten, lactose, sugars, and estrogen-rich foods, had significantly higher scores across all quality-of-life domains (physical functioning, psychological functioning, spiritual well-being, overall quality of life, social participation, and daily functioning) compared with non-adherent women, particularly those with strict adherence. The main barriers to implementation included limited awareness and the complexity of the dietary intervention. Krabbenborg et al.^8^ reported that, although women with endometriosis had lower overall diet quality (as assessed using the DHD-15 index^23^) compared with a healthy population, those who implemented specific dietary modifications perceived a notable improvement in pain-related symptoms, particularly after eliminating gluten, dairy, or soy, or increasing vegetable intake.

From an inflammatory perspective, Liu et al.^9^, in a cross-sectional study of a large population-based sample, found that a higher dietary inflammatory index (DII) was associated with an increased risk of endometriosis. Women with endometriosis tended to have more proinflammatory diets, and those in the highest DII tertile had a 57% higher risk compared with those in the lowest DII tertile. These findings underscore the potential role of an anti-inflammatory diet in disease prevention.

Xie et al.^3^ also found that adequate serum vitamin D levels were inversely associated with the prevalence of endometriosis, even after adjustment for multiple confounders, supporting the immunomodulatory and anti-inflammatory role of this micronutrient.

In contrast, Li et al.^10^, using Mendelian randomization, found no evidence of a causal relationship between genetically determined circulating antioxidant levels and the risk of endometriosis. This suggests that antioxidant supplementation may not be effective as a preventive strategy in healthy populations, although its potential therapeutic role in affected patients cannot be ruled out.

Regarding physical exercise, several studies highlight its potential beneficial effects on pain symptoms and quality of life. Artacho-Cordón et al.^24^ evaluated a multimodal therapeutic exercise program in women with endometriosis refractory to conventional treatments, reporting significant improvements in quality of life, reduced dyspareunia, decreased pain catastrophizing and increased pain thresholds in the lumbopelvic region. With respect to pelvic floor physical therapy, Del Forno et al.^5^,^25^ published two studies based on a randomized controlled trial. A significant reduction in superficial dyspareunia and chronic pelvic pain was observed following the intervention. However, no significant differences were found in urinary, bowel, or sexual functioning, although a trend toward improvement in constipation was noted.

Ensari et al.^1^ observed that women who engaged in regular physical exercise (at least three times per week) were more likely to experience a reduction in pain following exercise, suggesting an analgesic effect associated with habitual physical activity. In contrast, Sachs et al.^15^ compared physical activity levels between women with and without endometriosis and found that those affected engaged in less exercise and lower levels of daily physical activity, even after adjusting for variables such as dysmenorrhea, depression, and use of hormonal contraceptives, which may reflect barriers related to pain or fatigue.

Finally, Tennfjord et al.^14^, in a qualitative study, reported that participants in a supervised exercise program, including strength training and pelvic floor muscle training, perceived exercise as a safe and empowering tool for self-management of the disease and for enhancing social support.

**Table 3:** Studies included in the systematic review

Study	Country	Subject	Study design	Methodological quality and risk of bias	Sample size	Objectives	Intervention	Comparison	Results
Xie et al. 2024^3^	USA	Diet	Cross-sectional observational	Weak	3232	To examine the association between serum vitamin D levels and the presence of endometriosis	None	Comparison between women with adequate vs insufficient 25-hydroxyvitamin D3 levels	A significant inverse association was found between adequate vitamin D levels and risk of endometriosis (OR 0.73; 95% CI 0.54–0.97). No significant differences were observed in carbohydrate intake or vitamin C intake between groups.
Li et al. 2024^10^	China	Diet	Cross-sectional observational	Moderate	77,257	To assess the association between genetically determined circulating antioxidant levels and endometriosis risk	None	None	Absolute circulating antioxidant levels (retinol, β-carotene, lycopene, vitamin C) were not significantly associated with endometriosis risk (OR 0.62–1.01). Circulating antioxidant metabolites (γ-tocopherol, α-tocopherol, retinol, vitamin C) also showed no significant associations per unit increase (OR 0.91–1.04)
van Haaps et al. 2023^4^	Netherlands	Diet	Cross-sectional observational	Weak	211	To assess whether adherence to an endometriosis diet improves quality of life (“My Positive Health” questionnaire) in women with endometriosis	None	Comparison by level of adherence (strict, moderate, none)	Women adhering to the diet reported better quality of life than non-adherent women, with significant differences in total quality-of-life scores (1.9 vs 2.3), symptoms (2.1 vs 2.5), and social functioning (1.8 vs 2.2). Women who followed the diet also experienced fewer gastrointestinal (2.0 vs 2.4) and menstrual symptoms (2.2 vs 2.6), with all comparisons showing statistically significant differences (p < 0.05).
Ghasemisedaghat et al. 2023^2^	Iran	Diet	Case-control	Moderate	317	To examine the effects of a fertility diet on endometriosis.	None	107 cases with endometriosis vs 210 controls matched by age and other factors	Women with higher fertility diet scores had lower odds of developing endometriosis (OR 0.44, 95% CI: 0.27–0.71, p = 0.001 in the crude model; OR 0.46, 95% CI: 0.23–0.90, p = 0.022 in the adjusted model). Favorable associations were identified between the fertility diet and intake of plant protein and multivitamins, as well as unfavorable associations with intake of animal protein, heme iron, and glycemic load.
Liu et al. 2023^9^	USA.	Diet	Cross-sectional observational	Weak	3,410	To examine the association between dietary inflammatory index (DII) and endometriosis risk	None	Comparison between high vs low Dietary Inflammatory Index (DII)	Higher inflammatory index was associated with a 57% higher prevalence of endometriosis, particularly among non-obese, non-diabetic, hypertensive women, oral contraceptive users, and non-nulliparous women. In adjusted models, the ORs for women in the DII third quartile compared with those in the DII first quartile ranged from 1.55 to 2.25, depending on subgroup characteristics.
Krabbenborg et al. 2021^8^	Netherlands	Diet	Cross-sectional observational	Weak	157	To identify the dietary patterns followed by women with endometriosis and determine whether they perceive any effects on their disease.	None	None	Women with endometriosis reported symptom improvement following dietary modifications implemented after diagnosis. The proportion of participants reporting symptom improvement after eliminating specific foods was as follows: gluten (75%), dairy (70%), and soy (65%). Additionally, 80% of participants reported symptom improvement after increasing vegetable intake, and 60% reported improvement after increasing fish consumption.
Tennfjord et al. 2024^14^	Norway	Physical exercise	Qualitative	Moderate	41	To explore how physical activity and pelvic floor muscle training benefit women with endometriosis.	A four-month program of supervised weekly group training and home-based individual sessions (3-5 sessions per week).	Qualitative comparison pre-post intervention	Training women in physical activity and pelvic floor muscle exercise contributed to an improved perceived quality of life. The individualized and supervised nature of the program was positively valued, with participants reporting reduced fear of exercise.
Sachs et al. 2023^15^	Switzerland, Austria, Germany	Physical exercise	Cross-sectional observational	Moderate	920	To assess physical activity levels in women with and without endometriosis	None	Compares a group of women with surgically confirmed endometriosis with an age-matched group without suspected diagnosis.	Women with endometriosis engaged in fewer weekly hours of moderate (48.5% vs 52.1%) and vigorous physical activity (22.3% vs 25.4%), even after adjustment for confounding variables. The proportion of sedentary women was higher in the endometriosis group (29.2% vs 22.5%). Endometriosis, dysmenorrhea, and depression were associated with lower levels of physical activity.
van Haaps et al. 2023^21^	Netherlands	Physical exercise	Quasi-experimental	Weak	62	To evaluate whether the dietary intervention reduces pain and improves quality of life over six months	Low-FODMAP diet or endometriosis-specific diet.	Compares intervention group with control group and baseline values.	A significant reduction in pain was observed in the intervention group, with no changes in the control group. Participants reported less deep dyspareunia (mean difference −1.15) and less abdominal bloating (mean difference −0.99) compared with the control group, with statistically significant differences. Women who followed the diet showed significant improvements in 6 of 11 EHP-30 domains (pain, control/powerlessness, emotional well-being, self-image, work, and sexual functioning), with no changes observed in the control group.
Artacho-Cordón et al. 2023^24^	Spain	Physical exercise	Randomized controlled trial	Moderate	31	To evaluate the effectiveness of a supervised therapeutic exercise program in reducing pain, improving quality of life, and addressing lumbopelvic dysfunction in women with endometriosis.	Eight-week supervised, progressive, and individually tailored exercise program.	Standard medical treatment	The program was highly satisfactory and well tolerated. Significant and sustained improvements at one year were observed in quality of life, pain, strength, stability, and muscle mass. Dyspareunia was reduced in 90% of women who adhered to the instructions.
Del Forno et al. 2023^5^	Italy	Physical exercise	Randomized controlled trial	Moderate	30	To evaluate the effects of pelvic floor physiotherapy on urinary, bowel, and sexual function in deep infiltrating endometriosis	Five individual sessions of pelvic floor physical therapy.	No intervention; standard gynecological care.	Regarding pain, no statistically significant differences were observed between the intervention and control groups compared with baseline (p = 0.665). No significant differences were found between the two groups in urinary, bowel, or sexual function; however, women in the intervention group showed a trend toward improvement in constipation symptoms.
Ensari et al. 2022^1^	38 countries	Physical exercise	Cross-sectional observational	Weak	1009	To investigate the association of daily physical exercise with pain symptoms in endometriosis.	None	None	A linear model was used to analyze the relationship between previous-day exercise and daily pain, with habitual exercise frequency functioning as moderator. Habitual exercise frequency showed a small but statistically significant moderating effect (rate ratio: 0.96 for total pain score; pain score difference coefficient: -0.14; p < 0.05). As habitual exercise frequency increased, the effect of previous-day exercise on daily pain became more favorable. Participants with lower frequency or no regular exercise reported higher pain levels and greater increases in pain intensity following exercise.
Del Forno et al. 2021^25^	Italy	Physical exercise	Randomized controlled trial	Moderate	30	To evaluate pelvic floor muscle training on urinary, bowel, and sexual function in deep infiltrating endometriosis	Five individual pelvic floor sessions	No intervention; standard gynecological care.	The intervention group showed a mean reduction of 3 points in superficial dyspareunia on the numeric rating scale (IQR −4, −2), with no change in the control group (p < 0.01). A statistically significant difference was also observed in the change in chronic pelvic pain, with median values of 0 (IQR −2, 0) in the intervention group and (IQR 0, 1) in the control group.

## Discussion

The results of this systematic review suggest that both physical exercise and dietary interventions may play a relevant role in reducing symptoms and improving quality of life in women with endometriosis. Several studies report significant benefits in terms of reductions in pelvic pain and dyspareunia, as well as improvements in physical and psychological well-being, particularly when structured therapeutic exercise programs or anti-inflammatory diets are implemented.

Regarding diet, the analyzed studies show heterogeneous approaches, with elimination of potentially proinflammatory foods, such as dairy products and red meat, being the most common approaches. These restrictions have demonstrated greater effectiveness in reducing symptoms, particularly pain, compared with the isolated use of supplements^3^,^4^,^9^. Dietary modifications appear to benefit primarily patients with established disease, without clear evidence of a significant preventive role, and show greater effectiveness when combined with conventional treatments^10^,^26^. Anti-inflammatory diets may improve symptom control by reducing inflammation, oxidative stress, and cellular proliferation^27^,^28^.

A major challenge is treatment adherence, which is often affected by the psychological burden of the disease and may reduce motivation to adopt new habits^29^. Additionally, there is still a lack of consensus regarding which foods should be eliminated, despite the increasing recommendation of endometriosis-specific diets^4^,^30^.

Regarding physical activity, women with endometriosis tend to engage in less exercise due to symptom-related interference with daily life, which limits their ability to engage in physical activity^1^,^7^,^14^,^15^,^29^. However, when exercise is incorporated, benefits in symptom reduction are observed, particularly among women who have undergone surgical treatment^5^. Up to 42% use physical activity as a self-management strategy, although many lack adequate training^14^.

The literature indicates that physical activity increases pain tolerance, modulates the inflammatory response, and provides psychological benefits, such as improved self-esteem and a greater sense of control over health, in addition to preventing comorbidities associated with physical inactivity^1^,^14^,^15^,^31^. Physical activity may act through multiple mechanisms, including activation of endogenous pain inhibition pathways, reduction of proinflammatory cytokines, improved sensitivity to estrogen and progesterone, reduction of stress and chronically elevated cortisol levels, as well as improved insulin sensitivity, increased sex hormone-binding globulin (SHBG), and reduced circulating estrogen levels^16^,^17^.

When considering lifestyle changes, the economic factor is relevant, as many women lack access to specialized healthcare professionals in the private sector. In this context, nurses can play a key role as educators and promoters of healthy behaviors by providing guidance on care and self-care, while nutritionists and physical therapists are essential for a comprehensive approach, although their availability within healthcare services remains limited^4^,^30^.

For healthcare professionals, the available evidence does not support making specific recommendations for or against non-medical interventions, including physical activity and dietary modifications, due to the low methodological quality of the studies, inconsistent results, and potentially undefined adverse effects^32^,^33^. Hormonal pharmacological treatment remains the cornerstone of endometriosis management, controlling endometrial tissue proliferation, alleviating pain, improving fertility and reducing bleeding^7^. However, healthcare professionals should discuss with patients the possibility of incorporating non-pharmacological strategies as part of a comprehensive, person-centered approach, prioritizing shared decision-making, as these interventions may represent a beneficial option despite the existing uncertainty^7^,^34^-^36^. Exercise and physical activity may be considered components of a healthy lifestyle, although high-quality clinical trials demonstrating their specific effectiveness on disease symptoms are lacking^37^. Therefore, current clinical guidelines and professional societies do not recommend any specific non-medical intervention for endometriosis management, highlighting the need for further high-quality research in this field^32^,^33^,^36^,^37^.

This systematic review provides a comprehensive analysis of the recent literature on diet and exercise in women with endometriosis. Key strengths include a broad search strategy, inclusion of studies with diverse designs (randomized controlled trials, observational studies, and qualitative research), and systematic assessment of methodological quality and risk of bias using tools such as EPHPP and COREQ. This approach provides a comprehensive overview of the current state of the evidence, identifying consistent findings as well as areas of uncertainty.

When interpreting the results, several limitations should be considered. This systematic review included a relatively small number of studies, which may reflect the need for further research in this area. In addition, the protocol was not registered a priori, which may affect transparency. Methodological diversity among the included studies, heterogeneity in the interventions applied, and the use of different instruments to assess clinical outcomes hinder comparability across studies and reduce the external validity of the findings. Many studies included small sample sizes, nonrandomized designs, and a limited number of prospective studies, which increases the risk of bias and limits the ability to establish clear causal relationships. Publication bias cannot be ruled out, as studies with null or negative results may not have been published.

Future research should focus on multicenter randomized controlled trials with larger sample sizes and long-term follow-up, incorporating standardized measures of clinical outcomes and further elucidating the underlying pathophysiological mechanisms, thereby enabling the development of evidence-based recommendations.

## Conclusions

Current evidence suggests that lifestyle factors, such as diet and physical activity, may influence symptoms and quality of life in women with endometriosis. Non-pharmacological interventions, including regular physical exercise and the adoption of anti-inflammatory diets, may help reduce symptom severity and improve quality-of-life markers. However, further high-quality research is needed to establish robust evidence for this therapeutic approach and its long-term benefits.
